# Export Competitiveness of Agri-Food Sector during the EU Integration Process: Evidence from the Western Balkans

**DOI:** 10.3390/foods11010010

**Published:** 2021-12-21

**Authors:** Bojan Matkovski, Stanislav Zekić, Danilo Đokić, Žana Jurjević, Ivan Đurić

**Affiliations:** 1 Department of Agricultural Economics and Agribusiness, Faculty of Economics in Subotica, University of Novi Sad, 24000 Subotica, Serbia; stanislav.zekic@ef.uns.ac.rs (S.Z.); danilo.djokic@ef.uns.ac.rs (D.Đ.); zana.jurjevic@ef.uns.ac.rs (Ž.J.); 2 Department of Agricultural Markets, Leibniz Institute of Agricultural Development in Transition Economies (IAMO), 06120 Halle (Saale), Germany; duric@iamo.de

**Keywords:** agri-food, competitiveness, European Union, Western Balkans

## Abstract

Trade agreements with the European Union (EU) and Central European Free Trade Agreement (CEFTA) significantly influenced the liberalisation of agri-food products in Western Balkan (WB) countries. In all Western Balkan countries, there has been an intensification of the trade of agri-food products and a partial change in the regional and commodity structures of trade. This paper aims to identify comparative advantages of agri-food sectors and consider its tendencies during the EU integration process. Additionally, this paper will discuss some opportunities for improvement of the export positions of agri-food products. In that context and based on the literature review, the indexes of revealed comparative advantages and its modified version will be used as a main method for analysis in this research. Results showed that all Western Balkan countries, except Albania, have comparative advantages in exporting agri-food products. It is evident that Serbia has the highest level of comparative advantages in this sector. Moreover, this paper suggests that all countries should aim to provide the best possible positions for their agri-food products during pre-accession negotiations for EU membership and take the necessary steps towards increasing the level of competitiveness in the common EU market.

## 1. Introduction

The current policy of the Western Balkan (WB) countries, characterised by integration with the international market, brings numerous changes in the agri-food sector. As the countries of the WB have been in the process of adjusting the economic system to the rules of the European Union (EU) for a long time, there have been numerous changes in the market of agri-food products, which have had implications for their competitiveness [1,2]. Agriculture is an important sector of the national economy in all WB countries, contributing to economic and social stability [2]. The role of agriculture in WB countries is greater than the average for EU countries and agriculture is characterised by issues of unbalanced sectoral production, fragmented structure of agricultural holdings, relatively low yields, unfavourable export structure as well as poor food hygiene and quality control [3].

Market liberalisation in the WB was created by signing trade agreements with EU countries and signing a regional CEFTA agreement and other bilateral agreements. In the context of market liberalisation, significant results have been achieved in the foreign trade of agri-food products of the WB in the last decade, but export performances of the WB are significantly worse than in the EU countries [4]. One of the main causes, among others, is the low level of agricultural productivity [5]. Under the influence of the reached agreements on free trade, there was a partial change in the orientation of foreign trade of agri-food products of the WB and intensification of foreign trade flows with EU countries and CEFTA countries [6,7]. In that context, it is necessary to analyse the significance and intensity of foreign trade of agri-food products of these countries. Therefore, the main objective of this research is to identify comparative advantages of agri-food sectors of WB and consider their changes under integration processes. It is obvious that one of the primary aims of the WB countries is the quickest possible accession to the EU.

Defining competitiveness at the macro level is not an easy task given the fact that there is no universally accepted approach to defining it in the literature. Competitiveness at the macro level in the literature is most often based on superior product performance and the economy’s ability to be based on the production of highly productive products that can generate a high level of real income [8]. The World Economic Forum (WEF) definition is currently the most widely accepted. It views competitiveness as a set of institutions, policies, and factors that determine the level of productivity of the state [9]. However, the development of the concept of competitiveness at the macro level cannot be separated from the theories of trade relations, which suggests that national competitiveness is based on comparative advantage [10]. Although competitiveness and comparative advantages are sometimes seen as synonyms, it is necessary to make a distinction between them. Namely, competitive advantages are based precisely on comparative advantages, but many other factors determine the competitiveness of a country [11]. Since there is a limited number of papers in the literature dealing with the competitiveness of the agri-food sector in the WB, this research will fill this gap. In addition, the originality of this research is reflected in the relatively innovative concept for identifying the level of competitiveness and opportunities that affect the creation of export positions in the market of agri-food products.

The paper is organised as follows. After the introduction to the topic, the literature review is developed, as a theoretical basis for this research. The material and methods, which are used in this research are presented in the third section, while results based on these methods are presented in the fourth section. Presented results are elaborated together with discussion, where opportunities for the agri-food sector development are noted. The conclusion section includes policy implications (theoretical and practical) and considerations for future research on this topic.

## 2. Literature Review

Foreign trade liberalisation induced by international economic integrations also leads to significant changes in the level of competitiveness. Numerous authors have examined the changes in foreign trade in the global or regional market through the index of comparative advantages. For example, authors Bojnec and Ferto [12] analysed export competitiveness of agri-food products on international markets for major 23 countries, contributing to 60% of global agri-food trade, and concluded that most of them have comparative advantages. In contrast, export specialisation by country was found for a smaller number of agri-food products with comparative advantages. The same authors in another research [13] investigated drivers of the duration of comparative advantages of agri-food products in the EU and concluded that larger trade costs negatively influenced the duration of comparative advantages, while the level of economic development, the sise of the country, the agri-food export diversification and being a new EU member state, positively influenced it. Particularly interesting for the countries of the WB is the research of the effects of countries joining the EU, “new” member states, which shows the positive impact of EU accession on trade intensification, while almost all countries had a decrease in the comparative advantages of agri-food products after accession [14].

Research on the export competitiveness of agri-food products which include the WB region are rare. Research that focused on agri-food export performances of WB countries indicated that all countries of this region, except Albania, have comparative advantages in exporting these products, while export performances are lower than in EU countries [4]. Research by authors Matkovski et al. [1] focused on agri-food competitiveness in Southeast Europe and its determinants. These authors concluded that all Southeast Europe countries have comparative advantages in exporting these products on the global market (except Albania) and indicated the influence of partial productivities in agriculture on level of comparative advantages. Other research referred to the level of competitiveness of the processed food sector of the Danube region countries [15]. This research indicated that development of agri-food trade could have an important role in faster economic development.

On the level of the country of WB, there is some research in the previous literature. For example, research on Serbia indicated a high level of comparative advantages of the agri-food sector on global [16] and regional (WB) [17] markets. Another study on the Vojvodina region focused on the agribusiness sector and its possibilities in export on emerging markets [18]. This research showed that the situation in export from this region is better in plants than in livestock production and indicated the role of agricultural policy that can encourage more intensive production. The benchmark for setting future agricultural policy in WB countries is the Common Agricultural Policy (CAP) of the EU, so the pressure of EU negotiations and pre-accession support will positively influence the adaption of national agricultural policies to the CAP [19]. In recent years, there is some progress in aligning with EU requirements, but the structure of support is not favourable and is not in line with the EU [2]. Modifications of agricultural policy that could influence the competitiveness of agri-food products will be connected with the EU negotiation process. Previous literature showed that WB countries could not cope with competitive pressures with more developed countries. Furthermore, when the EU integration process is concerned, previous research indicates that better international economic positions involve North Macedonia and Montenegro than other candidates and protentional candidates for EU membership [20].

Based on a systematic review of the literature, Mizik [21] identified three significant factors for higher agri-food trade competitiveness:

- The supportive legislation and (trade) policy;
- Higher value-added/more sophisticated goods;
- Efficient and profitable production.

Some of the most important drivers of competitiveness are related to the knowledge economy. The use of knowledge in economic activities generates higher value-added goods, thus increasing the chances of success in this competitive and globalised world economy [22]. In modern global trends, new factors have emerged that affect competitiveness, both at the enterprise and state levels. According to Laitsou [23], the global economy as a framework is significantly related to the digital economy nowadays, while even traditional economic sectors (e.g., agriculture) implement increasingly more digital aspects, at least in developed economies. As a result, digital competitiveness is gaining increasing attention as a source of competitive advantage at the business and national economy levels. Also, Li et al. [24] showed market value in improving an enterprise’s green technological innovation ability. They concluded that if the enterprise is focused on green technological innovation inputs and outputs, it is more equipped to meet the green consumption demands from its consumers. As a result, its competitive advantage is enhanced through product differentiation.

To the best of our knowledge, there are no studies in literature analysing comparative advantages of the agri-food sector of all WB countries using indices of comparative advantages as in this paper, hence this research will contribute to filling this gap.

## 3. Material and Methods

One of the most suitable approaches for measuring competitiveness at the macro level is the tendencies in foreign trade flows [8]. Based on a systematic literature review, author Mizik [21] concluded that the most commonly used indices in the international literature are revealed comparative advantages (RCA) and its modified versions. The index of revealed comparative advantages was established by the author Balassa [25]:

(1) $${\mathrm{RCA}}_{\mathrm{ij}}=\frac{\frac{X_{\mathrm{ij}}}{X_{\mathrm{it}}}}{\frac{X_{\mathrm{nj}}}{X_{\mathrm{nt}}}}$$

where is: X—export; i—country; j—section or division; t—total export; n—group of exporting countries.

The existence of comparative advantages is indicated when RCA > 1. When the value of the RCA is greater than 3, the comparative advantages are strong, the values of the index of RCA between 2 and 3 indicate significant, while the values of RCA between 1 and 2 imply satisfactory comparative advantages. The traditional RCA index is widely used in the literature for the analysis of comparative advantages [for example [4,16,26,27,28]].

The RCA index is often criticised in the literature, due to it not considering the effects of countries’ economic policies. In addition, its point of view is asymmetric because it takes into account only export flows. Because of this, numerous modifications of this index have been developed. For example, Vollrath [29] developed a new specification of the revealed comparative advantages in the form of Relative Trade Advantages (RTA), which are calculated as the difference between the Relative Advantages of Export (RXA) and the Relative Advantages of Import (RMA):

(2) RTA=RXA−RMA

where is:

(3) RXA=RCA

And

(4) $$\mathrm{RMA}=\frac{\frac{M_{\mathrm{ij}}}{M_{\mathrm{it}}}}{\frac{M_{\mathrm{nj}}}{M_{\mathrm{nt}}}}$$

where is: M—import, i—country; j—section or division; t—total import; n—group of importing countries.

In contrast to RCA, the RMA less than 1 indicates the existence of comparative advantages. When RTA is concerned, if it is positive, that means the existence of comparative advantages of a particular section or division. Vollrath [29] highlighted the logarithmic values of RXA (ln RXA) as well as Revealed Competitiveness (RC) as two additional measures of comparative advantage:

(5) RC=lnRXA−lnRMA

When RC > 0, these sections or divisions have comparative advantages. According to Vollrath [29], RC is preferred, given that the balance of supply and demand is covered by the index and can provide better coverage of the comparative advantages of the analysed sections in a particular country. In the literature, modified indices are preferred over the traditional RCA index because they consider both exports and imports and eliminate double counting of countries and products, which can lead to insufficiently reliable calculations. These indicators have been used in numerous analyses of the competitiveness of specific sections, divisions, or products [for example [30,31,32]. The authors Balance, Forstner and Murray [33] suggest using specific statistical tests to see to what extent different indices of comparative advantage are consistent when assessing comparative advantage. Namely, these tests indicate the connection of different indicators when identifying comparative advantages. Analysing different indices of the comparative advantages, a comparative analysis is enabled in order to better overview potentials in comparative advantages of agri-food products of the Western Balkan countries.

An alternative measure of comparative advantage that is often used in the literature to express comparative advantage at lower levels of aggregation is the Lafay Index (LFI) [34]:

(6) $${\mathrm{LFI}}_{\;j}^{i}=100\;\left(\frac{x_{\;j}^{i}-m_{\;j}^{i}}{x_{\;j}^{i}+m_{\;j}^{i}}-\frac{\sum_{j=1}^{N}\left(x_{\;j}^{i}-m_{\;j}^{i}\right)}{\sum_{j=1}^{N}\left(x_{\;j}^{i}+m_{\;j}^{i}\right)}\right)\frac{x_{\;j}^{i}+m_{\;j}^{i}}{\sum_{j=1}^{N}\left(x_{\;j}^{i}+m_{\;j}^{i}\right)}$$

where is: x—export, m—import, i—country; j—section or division; t—total exports; n—country (group of countries) from which it is imported. When LFI > 0, it means the existence of comparative advantages; the higher the value of this index means the higher the level of specialisation of a certain country in the trade of the observed products. Numerous authors have used LFI to measure the comparative advantages of a sector or product [for example, [1,35,36]]. The advantage of LFI over the RCA index is reflected in a better picture of the foreign trade performance of the analysed product or sector because it considers exports and the side of imports. Thus, LFI stands out in the literature as a better method, bearing in mind that it provides a more comprehensive analysis of the specific positions of an individual product within its participation in the foreign trade of a particular country. Additionally, LFI is used to eliminate the influence of cyclical factors that can affect the trade flows in the short run, so this index is focused on the bilateral trade relations between the analysed countries [37].

The analysis is based on data collected based on a publicly available UN Comtrade database [38]. The quality and availability of data condition the period of the research. It refers to the period that is key to the research goal, i.e., the period when the liberalisation of trade in agri-food products occurred, which impacted the competitiveness of agri-food products (2005–2019). The term agri-food products includes the following divisions and product groups classified according to the SITC methodology (Standard International Trade Classification)—Revision 4: 00; 01; 02; 03; 04; 05; 06; 07; 08; 09; 11; 12; 21; 22; 261; 263; 264; 265; 268; 29; 41; 42; 43 (Table A1). This research covers six countries in the WB region: five countries candidates and potential candidates for EU membership and Croatia as a EU member state. Although Croatia became a member of the EU in 2013, it is included in the analysis as a benchmark for the remaining countries of the WB region.

## 4. Results and Discussion

**Figure 1:**
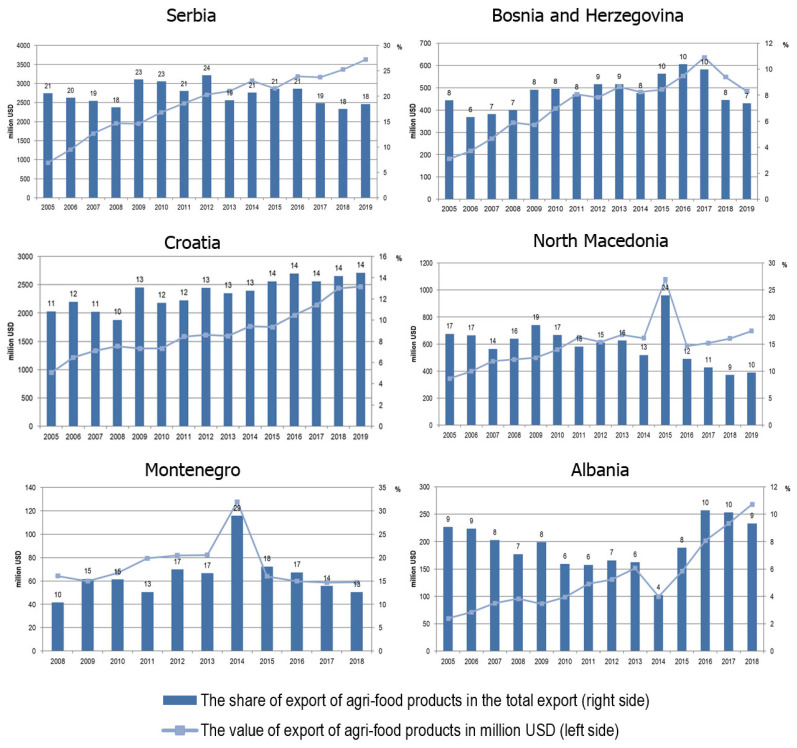
Export of agri-food products of the WB countries. Source: own research on basis of UN Comtrade [38].

The importance of the agri-food sector for the economy of the WB countries has been confirmed by the share of exports of these products in total exports. The largest share of agri-food products in total exports is evident in Serbia, where it was 20.4% on averagely in the analyzed period. The export of agri-food products is significant in exports and all other WB countries including in Montenegro (15.1%), North Macedonia (14.9%), Croatia (12.6%), Bosnia and Herzegovina (8.2%), Albania (7.7%); averages for the analysed period are shown in Figure 1. In absolute terms, the largest exporter of agri-food products in the WB region is Serbia, which exported these products on average in the value of over USD 2 billion annually in the analysed period, followed by Croatia, which exported these products on average about USD 1.6 billion annually in the analysed period. Regarding the dynamics of exports of these products, there was an increase in exports in the analysed period in all WB countries. The highest growth in exports of agri-food products was observed in Albania, where exports grew at an average annual rate of 10.4%; Serbia and Bosnia and Herzegovina also recorded high growth rates, where exports recorded an average annual growth rate of 8.4% and 7.5%, respectively.

The growth of exports of agri-food products is a consequence of the changed conditions of trade and the established liberalisation of exports with the EU and the CEFTA agreement within the countries of the WB. Although a crisis characterises the observed period, it can be seen that it did not negatively affect the export of these products, primarily due to their specific role in satisfying basic human needs. Namely, the use of products in daily nutrition cannot be easily reduced, and the agri-food sector does not have a significant negative reaction to the crisis [39].

In the structure of exports of agri-food products from the WB (Table 1), a section of the food and live animals is dominant (Section 0), which in most countries participates with over 50% of total exports of agri-food products. Analysis of the total exports of agri-food products within this section allows us to conclude that the most important export products are within the divisions of vegetables and fruit and cereals and cereal preparations.

The next section in terms of participation in the structure of exports of agri-food products in most WB countries is the beverage and tobacco section (Section 1). Within this section in most countries, it is exports of beverages at a higher level than exports of tobacco and tobacco manufactures, except in North Macedonia, where tobacco and tobacco manufactures are of greater importance in exports. The section of crude materials (Section 2) significantly participates in the structure of exports of agri-food products in Albania and Bosnia and Herzegovina, while the share of the section of animal and vegetable oils, fats and waxes (Section 4) is at a low level in the export structure of most WB countries. A slightly more significant share of the section of animal and vegetable oils, fats and waxes in the export of agri-food products is observed in Bosnia and Herzegovina and Serbia. It primarily refers to the export of products from the fixed vegetable fats and oils.

**Figure 2:**
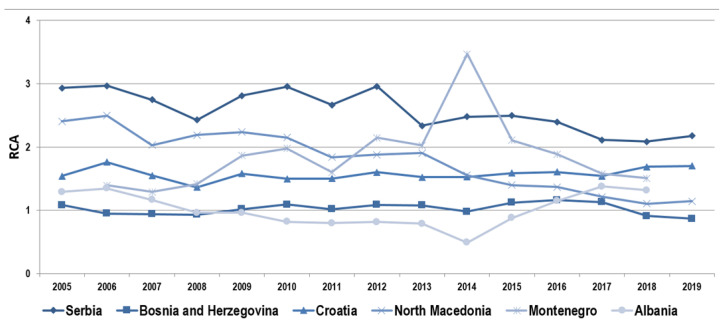
Revealed comparative advantages of the agri-food sector of the WB countries. Source: own research on basis of UN Comtrade [38].

Considering the values of the index of comparative advantages of agri-food products in countries of the WB (Figure 2), it can be concluded that all countries, except Albania, have comparative advantages in the export of these products in the analysed period. Analysing the differences in the comparative advantages of agri-food products by individual countries of the WB, it is obvious that Serbia has the highest level of comparative advantages in this sector, followed by North Macedonia, which also has significant comparative advantages, but in North Macedonia, the largest decline in comparative advantages in the analysed period was recorded.

Bosnia and Herzegovina, Croatia and Montenegro also have a satisfactory level of comparative advantages in the export of agri-food products, and positive tendencies in the movement of this index are noticeable in these countries. The largest oscillations are noticeable in Montenegro in the observed period, and considering that the value of exports of agri-food products and total exports is at a much lower level than in other countries, such tendencies are expected. Nevertheless, there was a drastic increase in comparative advantages in 2014, and in that year, a drastic increase in exports of meat and meat products from Montenegro was recorded. Croatia, which is the only EU member, has the greatest stability in the tendencies of comparative advantages, and to a certain extent it has stabilised the positions of its products on the international market. The worst situation is in Albania, which in most years did not achieve a satisfactory level of comparative advantages in exporting agri-food products to the international market. As already mentioned, negative tendencies in the movement of the index of revealed comparative advantages are noticeable in some countries. One of the reasons may be inadequate reactions to the improvement of competitiveness required by the world market in the conditions of regional and international integrations. Additional reasons can be found in the relatively poor export performance per hectare and employment in agriculture [4].

The modified indices of revealed comparative advantages show the dynamics of the movement of various indicators of comparative advantages of agri-food products in all WB countries (Table 2). It is important to note that these different trends are influenced by different ways of estimating comparative advantages, as some indices include import values of agri-food products, while some indices use only export values. Consistency among different indices is presented through correlation analysis.

When it comes to Serbia, it can be noticed that all indicators indicate strong comparative advantages. At the same time, in North Macedonia, there are significant comparative advantages of this sector on the international market. Analysis of modified indices of revealed comparative advantages in Bosnia and Herzegovina and Montenegro shows that only the traditional index of revealed comparative advantages (RXA = RCA) shows a satisfactory level of comparative advantages of agri-food products on the international market, and it is strongly correlated with the logarithmic version of this index. When it comes to Croatia, RXA and lnRXA indicate the existence of comparative advantages in the export of agri-food products, while when the analysis includes the import of these products, the analysis results show that since 2013 the comparative advantages of these products are weak, which is the moment of Croatia’s accession to the EU. The EU accession leads to significant policy changes and can certainly impact foreign trade flows, both positive if the country is adequately prepared for a highly competitive EU market, or negative if this preparation is lacking. Reasons for the weak competitiveness level of Croatia are unfavourable productional structure, lack of cooperation between producers, poor risk management, weak physical infrastructure of the market, and weak agri-food chains [40]. When it comes to the comparative advantages of the agri-food sector of Albania on the international market, the comparative advantages on the international market are practically the worst within the region of the WB.

**Figure 3:**
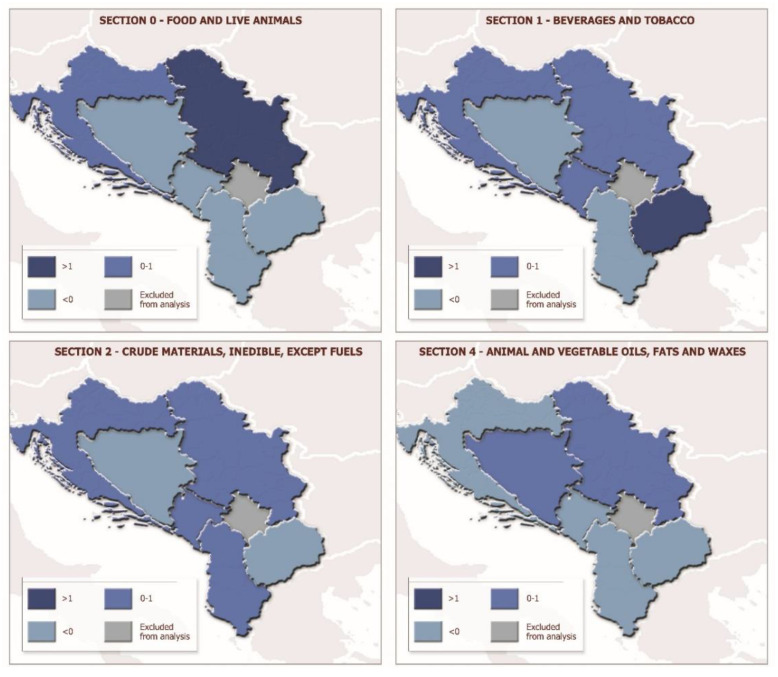
Revealed comparative advantages of sections of the agri-food sector of the WB countries (LFI). Source: own research on basis of UN Comtrade [38].

The LFI index (Figure 3) was used to calculate the comparative advantages at the level of individual sections of agri-food products. Considering the differences in comparative advantages in agri-food products by individual sections, it can be seen that the food and live animals section (Section 0), which for all WB countries averages over 60% of exports and 70% of imports of agri-food products, has strong comparative advantages only in Serbia, when the greatest comparative advantages are achieved by cereals and cereal preparations (Table 3). In Serbia, the largest part of Section 0 has comparative advantages on the international market, except for the division of fish, crustaceans, molluscs, and aquatic invertebrates and preparations thereof, as well as the division of coffee, tea, cocoa, spices and manufactures from them. Croatia also has comparative advantages of the entire Section 0, where only certain divisions of this sector have comparative advantages: fish, crustaceans, molluscs and aquatic invertebrates, and preparations thereof, sugars, sugar preparations, and honey, miscellaneous edible products and preparations, as well as cereals and cereal preparations. All remaining countries do not have comparative advantages for agri-food products from Section 0, the only exceptions being that some divisions that have comparative advantages, including North Macedonia sections of live animals and vegetables and fruit as well as in the Albania section of fish, crustaceans, molluscs, and aquatic invertebrates, and preparations thereof.

The beverages and tobacco section (Section 1) has comparative advantages on the international market in most WB countries; this section’s highest level of comparative advantages in the analysed period is recorded in North Macedonia, followed by Montenegro, Serbia, and Croatia. Within Section 1, beverages lead in relation to tobacco and tobacco manufactures in the level of achieved comparative advantages in Serbia and Montenegro, while the situation is opposite in North Macedonia and Croatia, where tobacco and tobacco manufactures have a higher level of revealed comparative advantages in relation to beverages. The crude materials section (Section 2) has comparative advantages on the international market in the majority of the WB countries, except North Macedonia and Bosnia and Herzegovina, while the animal and vegetable oils, fats and waxes section (Section 4) has comparative advantages only in Serbia and Bosnia and Herzegovina.

Despite the significant intensification of foreign trade under the influence of liberalisation and the existence of comparative advantages of agri-food products on the international market, there are numerous problems of the agri-food sector; therefore, eliminating these problems will be important in achieving better competitive positions on the international market. Namely, the technological level of the agricultural sector of the WB is at a lower level, which is a consequence of insufficiently innovative production structure. The livestock production is lagging behind the EU [41], so it is necessary to work on knowledge transfer in this area. Moreover, processing on the farm itself is underrepresented [42], and this type of adding value to the product would affect the improvement of the position of farms. In order to improve the production performance of agriculture, agricultural policy measures need to encourage more intensive agricultural production, which further aims to create a better basis for the development of the food industry. Improving competitiveness will not be possible without adapting to the high and growing demands of the developed EU market, especially when production standards are concerned. Apart from the state support, organisational leaders with their initiatives also have an important role in promoting the international standards for business operations in the function of raising the competitiveness level [43]. As the structure of farms in the majority of the WB countries is unfavourable [44], where small farms predominate, the appearance of producers on the EU market is very difficult. Therefore, all these countries aim to provide the best possible position for their agricultural producers during the pre-accession negotiations for EU membership and take the necessary steps towards increasing the level of competitiveness in the future common EU market. The crucial task will be strengthening the institutional capacity for better organisation of small agricultural producers, for example, in forms of cooperatives and clusters. In addition to institutional capacities, it is necessary to promote cooperatives in order to increase awareness of the benefits of such an organisation. Of course, adequate education of farmers is also needed.

In general, in the countries of the WB, the export performance of the agricultural sector is largely related to the production performance of agriculture, which is strongly determined by agro-ecological conditions for production [4]. These performances are the consequence of the relative extensiveness of the agricultural sector of these countries, as well as the weakness of the processing industry [45]. Full membership in the EU, as is the case with Croatia, has a twofold effect on the level of competitiveness. On the one hand, a higher level of support is available to the agricultural sector. On the other hand, the market is exposed to much greater competitive pressure. In this context, the example of Croatia after joining the EU is encouraging, with the fact that the food industry in this country is traditionally more successful than other countries in the WB, which is also shown by the most favourable export structure (Table 1).

There are some opportunities for the countries of the WB. For example, there is a noticeable trend of growing demand for organic food in developed countries. Končar et al. [46] showed statistically significant correlations between consumers’ decisions to purchase an organic food product and different indicators, such as the origin, composition of raw materials, freshness, price, taste, and place of sale, quality label and packaging. Adequate organic food labelling and institutional support are critical constraints to developing organic production in the WB. Also, the growth of domestic demand for these products would significantly accelerate the development of organic production. According to Radojević et al. [47], to boost demand, it is necessary to improve consumer knowledge about what organic production is and how products from this production system are appropriately labelled. This is crucial due to a positive impact of organic production on the environment and the sustainability of the food system [48].

Furthermore, there is a problem of dual quality food in the European market. Some recent research from Central and Eastern Europe showed that some agri-food products sold in their regions are of lower quality and less healthy than the same brands in Western Europe [49]. This can be the opportunity for some WB companies to complete market niches, i.e., gain the trust of these consumers, and are better positioned in the market of Central and Eastern European countries.

## 5. Conclusions

Based on the obtained results, it is possible to emphasise several conclusions:

- In the field of foreign trade, in all WB countries, there has been an **intensification of the trade of agri-food products** and a partial change in the orientation of the trade. In all countries of the WB, there is an increase in exports of agri-food products.
- In analysing the position of agri-food products on the international market, it is noticed that the **structure of exports is not particularly favourable**, considering that a large percentage of exports are crop products of lower processing, cereals and fruits and vegetables. In contrast, the share of livestock products is inadequate. In that context, a technological adjustment in livestock production is necessary to achieve a higher level of efficiency in production, which would result in a higher level of competitiveness in the international market.
- Considering the comparative advantages of agri-food products of the WB countries, it can be noticed that **all countries have comparative advantages on the global market, except Albania**. Serbia has the highest level of comparative advantages of this sector, followed by North Macedonia. The most unfavourable situation in terms of comparative advantages of agri-food products is in Albania, which in most years does not achieve a satisfactory level of comparative advantages in exporting these products to the international market.

The research results indicate changes in the level of comparative advantages, which is significant from both the macroeconomic and microeconomic aspects, and the results of this research have certain **policy implications**. Given that liberalisation also poses a threat to the agri-food sector, research results may indicate segments of this sector within which additional efforts are needed to lead to improved competitiveness. The research results could be useful for agricultural policymakers, in terms of creating more effective support to the agri-food sector, which would contribute to “favouring” domestic producers, and at the same time, increase competitiveness in the international market. This research has some **limitations**, as the specific situation of WB countries cannot provide general conclusions as distribution of the indices of comparative advantages may differ considerably across countries, thus comparative analysis can sometimes be problematic [26]. Moreover, the factors that affect competitiveness have not been included in the statistical analysis. As a result, **future research** can be directed towards a more detailed review of the competitive positions of specific parts of the agri-food sector and analysis of factors influencing changes in the competitiveness of agri-food products on the international, regional and EU markets.

## Figures and Tables

**Table 1:** Commodity structure of export of agri-food sector of the WB countries.

Divisions	Serbia	Bosnia and Herzegovina	Croatia	North Macedonia	Montenegro	Albania
**Section 0** **—Food and live animals**	**75.1%**	**66.8%**	**75.5%**	**56.9%**	**46.4%**	**69.5%**
Live animals	1.6%	0.7%	2.9%	0.4%	0.0%	0.6%
Meat and meat preparations	3.5%	8.6%	6.5%	5.4%	17.4%	1.2%
Dairy products and birds’ eggs	3.3%	10.2%	4.4%	2.1%	0.4%	1.4%
Fish (not marine mammals), crustaceans, molluscs and aquatic invertebrates, and preparations thereof	0.3%	2.6%	10.6%	1.1%	0.5%	31.4%
Cereals and cereal preparations	22.9%	11.0%	12.9%	10.4%	6.3%	2.2%
Vegetables and fruit	24.8%	16.7%	5.2%	29.7%	14.0%	29.4%
Sugars, sugar preparations and honey	6.3%	7.4%	9.2%	1.7%	0.5%	0.2%
Coffee, tea, cocoa, spices, and manufactures thereof	3.3%	3.8%	7.0%	2.5%	4.1%	1.5%
Feeding stuff for animals (not including unmilled cereals)	4.5%	2.6%	3.6%	0.2%	0.3%	1.3%
Miscellaneous edible products and preparations	4.6%	3.2%	13.3%	3.3%	2.9%	0.3%
**Section 1** **—Beverages and tobacco**	**13.3%**	**8.4%**	**14.7%**	**37.6%**	**43.3%**	**5.9%**
Beverages	7.6%	6.1%	8.1%	13.8%	39.5%	2.9%
Tobacco and tobacco manufactures	5.7%	2.3%	6.6%	23.9%	3.8%	3.0%
**Section 2** **—Crude materials, inedible, except fuels**	**5.6%**	**13.3%**	**7.5%**	**3.5%**	**7.8%**	**23.9%**
Hides, skins and furskins, raw	1.1%	11.5%	1.6%	1.0%	7.2%	6.3%
Oil-seeds and oleaginous fruits	3.1%	0.5%	4.6%	0.4%	0.0%	0.0%
Crude animal and vegetable materials, n.e.s	0.1%	0.2%	0.1%	0.2%	0.1%	0.1%
Silk, cotton, jute, vegetable textile fibres, wool and other animal hair	1.3%	1.1%	1.1%	1.9%	0.5%	17.5%
**Section 4—** **Animal and vegetable oils, fats and waxes**	**6.0%**	**11.4%**	**2.3%**	**1.9%**	**2.5%**	**0.7%**
Animal oils and fats	0.1%	0.0%	0.2%	0.0%	1.0%	0.0%
Fixed vegetable fats and oils, crude, refined, or fractioned	5.7%	11.4%	1.8%	1.8%	1.3%	0.7%
Animal or vegetable fats and oils, processed	0.2%	0.1%	0.4%	0.1%	0.2%	0.0%

Source: own research on basis of UN Comtrade [38].

**Table 2:** Revealed comparative advantages of agri-food sector of the WB countries calculated by different indicators.

Serbia				
Mean 2005–2019	2.57	1.65	0.94	1.02
Coef. of variation	12.3%	19.7%	13.4%	15.4%
*Correlation analysis*				
	RXA	RTA	ln RXA	RC
RXA	1			
RTA	0.9648	1		
ln RXA	0.9988	0.9671	1	
RC	0.7906	0.9235	0.7994	1
**Bosnia and Herzegovina**				
Mean 2005–2019	1.02	−1.26	0.02	−0.80
Coef. of variation	8.8%	−16.0%	431.0%	−13.6%
*Correlation analysis*				
	RXA	RTA	ln RXA	RC
RXA	1			
RTA	0.2532	1		
ln RXA	0.9993	0.2289	1	
RC	0.6474	0.9007	0.6282	1
**Croatia**				
Mean 2005–2019	1.57	0.12	0.45	0.08
Coefficient of variation	6.1%	138.3%	13.6%	136.5%
*Correlation analysis*				
	RXA	RTA	ln RXA	RC
RXA	1			
RTA	0.3533	1		
ln RXA	0.9986	0.3288	1	
RC	0.2942	0.9973	0.2699	1
**North Macedonia**				
Mean 2005–2019	1.91	0.27	0.61	0.13
Coef. of variation	28.2%	117.4%	49.9%	121.7%
*Correlation analysis*				
	RXA	RTA	ln RXA	RC
RXA	1			
RTA	0.6827	1		
ln RXA	0.9920	0.7115	1	
RC	0.6994	0.9925	0.7379	1
**Montenegro**				
Mean 2006–2018	1.87	−0.96	0.59	−0.44
Coefficient of variation	30.1%	−39.9%	44.0%	−38.0%
*Correlation analysis*				
	RXA	RTA	ln RXA	RC
RXA	1			
RTA	0.7194	1		
ln RXA	0.9846	0.6122	1	
RC	0.9116	0.9220	0.8599	1
**Albania**				
Mean 2005–2018	0.98	−1.10	−0.05	−0.76
Coef. of variation	23.9%	−32.1%	−531.1%	−31.4%
*Correlation analysis*				
	RXA	RTA	ln RXA	RC
RXA	1			
RTA	0.1928	1		
ln RXA	0.9858	0.1441	1	
RC	0.7154	0.8171	0.6878	1

Note: RXA > 1; RTA > 0; ln RXA > 0; RC > 0. Source: own research on basis of UN Comtrade [38].

**Table 3:** Index of revealed comparative advantages of divisions of agri-food sector of the WB countries (LFI).

Divisions	Serbia	Bosnia and Herzegovina	Croatia	North Macedonia	Montenegro	Albania
**Section 0—** **Food and live animals**	**4.63**	**−3.40**	**0.04**	**−1.12**	**−2.94**	**−2.61**
Live animals	0.10	−0.21	−0.08	0.00	−0.26	−0.24
Meat and meat preparations	0.14	−0.38	−0.27	−0.72	−0.47	−0.45
Dairy products and birds’ eggs	0.19	−0.10	−0.12	−0.26	−0.59	−0.16
Fish (not marine mammals), crustaceans, molluscs and aquatic invertebrates, and preparations thereof	−0.16	−0.06	0.36	−0.11	−0.14	0.60
Cereals and cereal preparations	1.99	−0.77	0.25	−0.06	−0.48	−1.35
Vegetables and fruit	1.65	−0.16	−0.52	1.17	−0.06	0.02
Sugars, sugar preparations and honey	0.50	−0.21	0.29	−0.26	−0.14	−0.32
Coffee, tea, cocoa, spices, and manufactures thereof	−0.16	−0.58	−0.05	−0.38	−0.33	−0.31
Feeding stuff for animals (not including unmilled cereals)	0.26	−0.38	−0.18	−0.19	−0.20	−0.13
Miscellaneous edible products and preparations	0.13	−0.55	0.37	−0.32	−0.27	−0.29
**Section 1—** **Beverages and tobacco**	**0.75**	**−0.93**	**0.39**	**1.76**	**0.79**	**−0.99**
Beverages	0.52	−0.69	0.15	0.56	0.83	−0.52
Tobacco and tobacco manufactures	0.23	−0.25	0.24	1.20	−0.05	−0.47
**Section 2—** **Crude materials, inedible, except fuels**	**0.22**	**−0.02**	**0.18**	**−0.07**	**0.21**	**0.58**
Hides, skins and furskins, raw	0.06	0.25	0.06	0.04	0.28	0.17
Oil-seeds and oleaginous fruits	0.17	−0.17	0.23	−0.07	−0.01	0.00
Crude animal and vegetable materials, n.e.s	−0.02	−0.04	−0.01	−0.03	0.00	−0.01
Silk, cotton, jute, vegetable textile fibres, wool and other animal hair	0.01	−0.06	−0.09	−0.02	−0.05	0.42
**Section 4—** **Animal and vegetable oils, fats and waxes**	**0.46**	**0.04**	**−0.07**	**−0.29**	**−0.09**	**−0.35**
Animal oils and fats	0.00	−0.01	0.00	−0.01	0.03	−0.03
Fixed vegetable fats and oils, crude, refined, or fractioned	0.45	0.11	−0.07	−0.26	−0.12	−0.32
Animal or vegetable fats and oils, processed	0.01	−0.06	0.00	−0.02	0.00	−0.01

Source: own research on basis of UN Comtrade [38].

## Data Availability

The datasets generated for this study are available on request to the corresponding author.

## Appendix A

**Table A1:** Agri-food products included in analysis according to SITC methodology.

Section/Divisions/Groups
**Section 0—** **Food and live animals**
00—Live animals
01—Meat and meat preparations
02—Dairy products and birds’ eggs
03—Fish (not marine mammals), crustaceans, molluscs and aquatic invertebrates, and preparations thereof
04—Cereals and cereal preparations
05—Vegetables and fruit
06—Sugars, sugar preparations and honey
07—Coffee, tea, cocoa, spices, and manufactures thereof
08—Feeding stuff for animals (not including unmilled cereals)
09—Miscellaneous edible products and preparations
**Section 1—** **Beverages and tobacco**
11—Beverages
12—Tobacco and tobacco manufactures
**Section 2—** **Crude materials, inedible, except fuels**
21—Hides, skins and furskins, raw
22—Oil-seeds and oleaginous fruits
29—Crude animal and vegetable materials, n.e.s
261, 263, 264, 265, 268—Silk, cotton, jute, vegetable textile fibres, wool and other animal hair
**Section 4—** **Animal and vegetable oils, fats and waxes**
41—Animal oils and fats
42—Fixed vegetable fats and oils, crude, refined, or fractioned
43—Animal or vegetable fats and oils, processed

Source: own presentation on basis of UNstats [50].
